# *P.I. Charter*: A Shiny Application for Collecting, Cleaning, Compiling, and Communicating Point‐Intercept Aquatic Plant Data

**DOI:** 10.1002/ece3.73779

**Published:** 2026-06-03

**Authors:** Alex W. Bajcz, Daniel J. Larkin, Michael R. Verhoeven, Raymond M. Newman, Jake R. Walsh, Abha Panda, Nicholas B. D. Phelps

**Affiliations:** ^1^ Department of Fisheries, Wildlife and Conservation Biology and Minnesota Aquatic Invasive Species Research Center University of Minnesota‐Twin Cities St. Paul Minnesota USA

**Keywords:** adaptive management, collaborative database, data interoperability, invasive species, lake ecology, macrophytes, point‐intercept surveys, R Shiny

## Abstract

Even when data needed to address complex, high‐priority ecological questions exist, when they are collected by many different entities for diverse purposes, they can be difficult to discover and unify. Web applications can address these challenges by serving as submission portals that clean and combine data programmatically. Furthermore, web apps can incentivize contributions—e.g., by providing public recognition and novel visualizations for reporting—while insulating users from needing to apply programming skills or interoperability practices. Here, we introduce *P.I. Charter*, an R Shiny web application that solicits, tidies, compiles, and shares point‐intercept (PI) aquatic plant survey data collected across Minnesota, USA by dozens of entities. We summarize the app's features and how these align with our goals regarding ease of use, accessibility, education, open access, and user experience. Since *P.I. Charter*'s launch, it has received thousands of visitors and hundreds of submissions. Familiar contributors can submit surveys in minutes, and satisfaction with the app is high. We present four case studies that show how the app and its database could be used to: (1) better plan and conduct future surveys, (2) strategize surveillance for new cryptic invasions, (3) investigate relationships between species occurrence and environmental factors, and (4) elucidate long‐term trajectories of plant community composition in lakes responding to global change processes and management. Apps like *P.I. Charter* could revolutionize data aggregation in contexts wherein large data volumes are gathered and stored disparately. For us, developing *P.I. Charter* was relatively straightforward, but we caution that using R Shiny to build a complex, science‐focused web application is not without challenges, especially with respect to achieving digital accessibility. Nonetheless, *P.I. Charter*'s capacity to make fuller use of hard‐earned data to address ecological questions has proven so valuable we wish to share our experiences so they may serve as a model for others.

## Graphical abstract

When ecological data are collected by many entities for different purposes, they can be difficult to locate and integrate; web applications can address this by standardizing submission, automating cleaning, and incentivizing contributions. We present *P.I. Charter*, an R Shiny app that compiles and shares point‐intercept aquatic plant survey data across Minnesota while prioritizing usability, accessibility, and open access. Since launch, the app has attracted substantial use and supports applications ranging from survey design to invasion surveillance and long‐term ecological analysis, though development required careful attention.

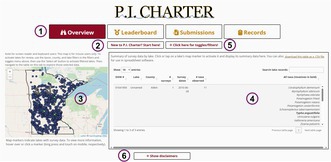

## 1 Introduction

Answering complex, pressing ecological questions—both theoretical and applied—increasingly demands voluminous, high‐quality data sets. Three examples prove the point: Delineating a species' range may require thousands of location records, if not magnitudes more (Welsh et al. 2013); confirming a species is endangered may hinge on long‐term monitoring across many systems (Borgelt et al. 2022); and evaluating management effectiveness requires robust treatment and reference data sets (Verhoeven, Larkin, et al. 2020). Without enough high‐quality data, many timely questions will remain unanswered.

Regrettably, such data often exist but are highly distributed. Monitoring—whether by community groups, researchers, nonprofits, businesses, governmental agencies, etc.—is typically conducted to fulfill a specific purpose for the collecting entity only. While the data generated by such efforts are often gathered to be *comparable* to those of others, *interoperability* (Wilkinson et al. 2016) is a rarer consideration. As such, large‐scale, disparately collected ecological data sets are rarely *combinable*, let alone combined. This fragmentation limits what questions can be answered and by whom; a researcher may be well‐positioned to analyze large data sets but lack the ability to collect or aggregate data of sufficient volume; the opposite might be true for monitoring groups.

One example of valuable but siloed ecological data is those from point‐intercept (PI) surveys, a technique for producing systematic, data‐rich accounts of lake macrophyte communities (Madsen and Wersal 2018). Broadly, a PI survey involves overlaying a lake's plant‐habitable (“littoral”) zone with a grid of points and sampling each intersection with a rake, recording the abundance of all taxa retrieved (Perleberg et al. 2016). In Minnesota, USA, thousands of PI surveys have been conducted since 2000 by governmental agencies, lake associations, park districts, watershed management districts, environmental consultancies, and others using comparable methods. Several neighboring states, including Wisconsin (Hauxwell et al. 2010), Michigan (Alexander 2022; Michigan Department of Environment, Great Lakes, and Energy 2022), and Iowa, have similar circumstances.

Despite their potential to answer pivotal questions (e.g., Verhoeven, Glisson, et al. 2020; Verhoeven, Larkin, et al. 2020), Minnesota's PI data remain fragmented. Though collected similarly, they are generally stored according to each entity's needs and workflows, generating files that often violate FAIR data principles (Wilkinson et al. 2016). For example, inconsistent missing‐value coding and multiple distinct data stored in the same cell are common. Merging even two such data sets “by hand” can take hours, even when both are internally consistent. Many organizations lack the capacity and/or incentive to adopt more FAIR practices, especially when no central entity exists to codify or mandate such practices. Thus, important questions answerable with a PI database, such as “How are lake plant communities being affected by climate change?” and “How do invasive aquatic plants affect native plant communities?” remain difficult to explore.

In these contexts, web applications can be the missing centralizer. Entities can access an app at their convenience to submit data. Using the submitter's inputs, the app can programmatically clean and standardize submissions and then analyze, visualize, and recontextualize the data in real time. Beyond making the data interoperable and usable by others, an app could improve the workflow of contributors by automating data cleaning, providing new outputs for reporting, and informing future survey efforts.

Here, we introduce *P.I. Charter* (available at z.umn.edu/PICharter), an R Shiny web application (Chang et al. 2026) that collects, standardizes, and publicly shares PI data, enabling communal access to a large, previously fragmented data set. Additionally, *P.I. Charter* explains PI survey methodology; tracks when, where, and by whom surveys have been conducted; and offers outputs relevant to data exploration, research, and management. Importantly, the app requires minimal oversight (< 5 h/mo, on average), reducing the maintenance burden on its host (the Minnesota Aquatic Invasive Species Research Center [MAISRC]).

We have three goals with this publication: (1) to describe *P.I. Charter* and how its design aligns with our vision (see *Design vision*); (2) to demonstrate the app's utility in answering critical questions; and (3) to argue that *P.I. Charter*'s design is usefully transferable to many other contexts. While Shiny apps are increasingly transforming data exploration (Jia et al. 2022), data manipulation (Gibbons et al. 2023), teaching (McGuire et al. 2022), analysis (Colomb and Winter 2021), science communication (Whitehead and Booker 2019), and decision support (Li 2020), their potential as data aggregation hubs remains underrealized (but see Kaufman 2020). *P.I. Charter's* capacity to leverage R's (R Core Team 2025) data‐wrangling capabilities to unify disparate data—an otherwise time‐intensive task—is perhaps its most salient feature. Given the rapid ecological change underway, apps like *P.I. Charter* could break down silos constraining progress on key questions.

## 2 Materials and Methods

### 2.1 Design Vision

We designed *P.I. Charter* with five core tenets:

- *Ease*: The app should be easy and intuitive to find and use. Contributors should be able to easily submit data without creating significant post‐processing demand. Users should be able to quickly access and understand information presented in the app.
- *Accessible*: The app should be usable by everyone, no matter their experience level or navigation devices.
- *Educational*: Users should come to value PI surveys as sources of lake plant community ecology data.
- *Open*: The app should not only collect and house data but enhance and share them, facilitating mutually beneficial exchange.
- *Rewarding*: Contributors should feel acknowledged for contributing, and noncontributing users should feel rewarded too.

*P.I. Charter*'s structure maps to these design principles through the inclusion of four tabs as described below.

### 2.2 Overview Tab

**FIGURE 1:**
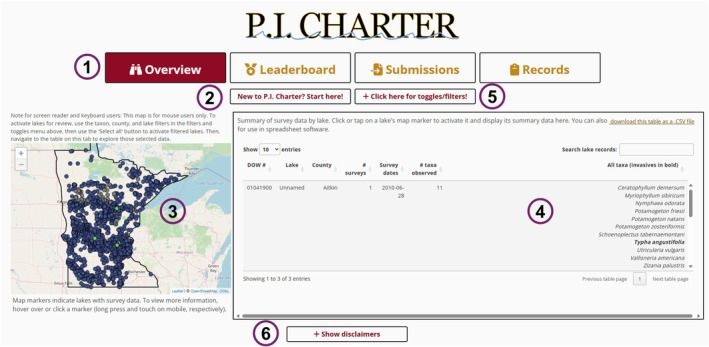
“Overview” tab of *P.I. Charter*. (1) Tab navigation buttons; (2) Menu (open on launch) containing background, tutorials, and resources for directed learning; (3) Map showing one marker per lake in the database; (4) Table displaying summaries for selected lakes; (5) Menu of inputs for filtering and interacting with the map and table; and (6) Disclaimers menu.

The “Overview” tab of *P.I. Charter*, open on launch, is designed to educate users on the app and on PI surveys and to support accessible, high‐level exploration of the database (Figure 1). It has four primary features: a *leaflet* map (Cheng et al. 2025) showing the spatial distribution of our database, a DataTables (*DT*) table (Xie et al. 2024) displaying lake‐level summaries, and two menus enabling targeted browsing and learning.

On launch, the map displays all lakes in the database. When a user activates a marker (by clicking it), that lake's summary data are loaded. These include how often and when each lake was surveyed and which and how many taxa have been observed to date. The map can be panned and zoomed, and the table can be sorted, searched, and filtered.

Two menus support the tab's educational and exploratory mission. The first, open on launch, provides tutorials, background, and external resources so users understand the purpose, methodology, and intentions of PI surveys, *P.I. Charter*, and its “Overview” tab. It also provides language entities can adopt to incorporate data‐submission requirements into PI survey contracts. The second menu offers inputs for interacting with and filtering the map and table. Users can filter the map by taxon, county, and/or lake, facilitating finding specific records. Buttons allow users to activate all markers or to reset selections. A toggle is available that switches between common and scientific names, supporting a broader range of users. Notably, the taxon selector allows users to compare geographic distributions of taxa and identify lakes where two or more taxa co‐occur. Finally, users can download the table for offline use.

### 2.3 Submissions Tab

**FIGURE 2:**
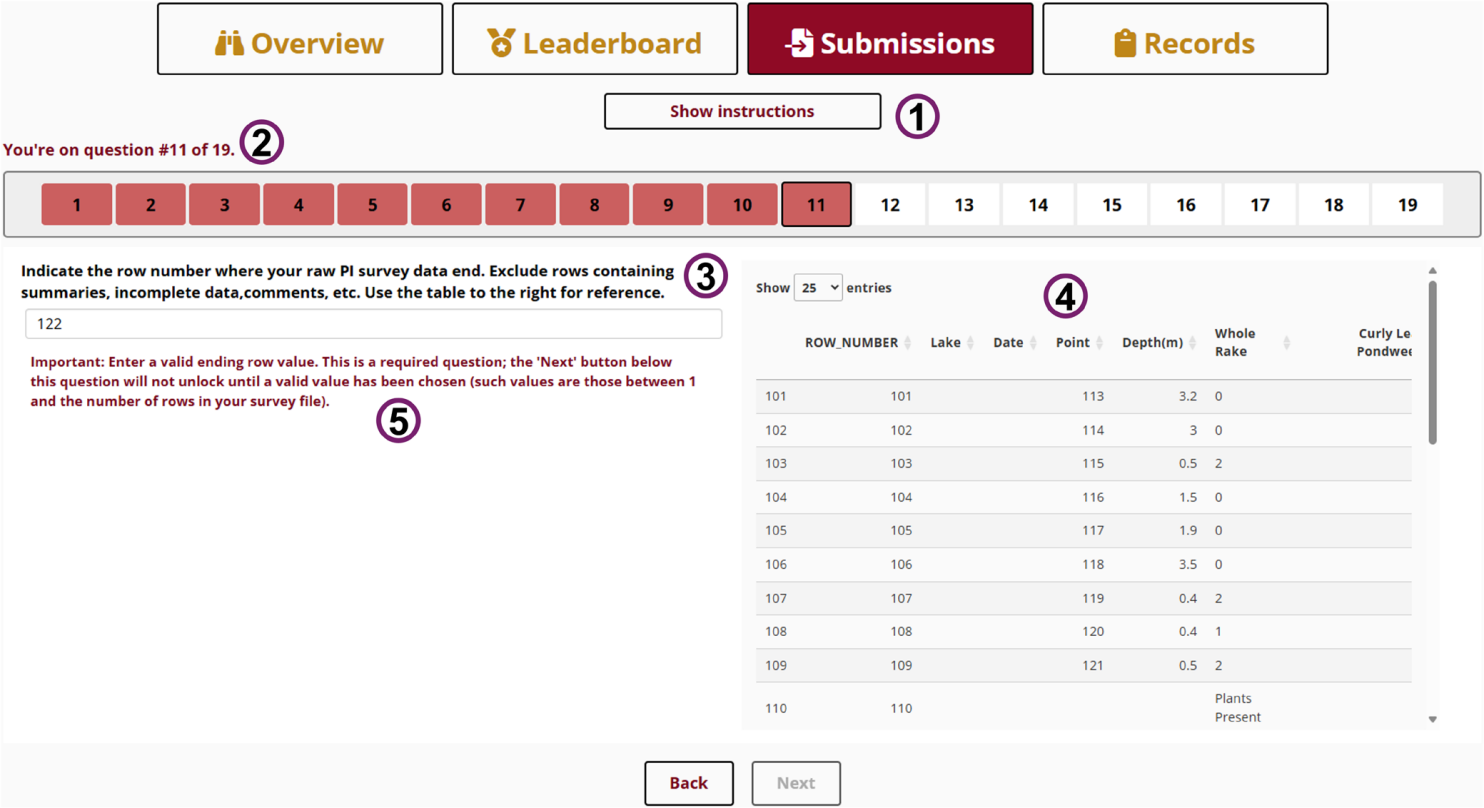
“Submissions” tab of *P.I. Charter*. (1) Tutorials menu for the submission process. (2) Progress through the 19‐question form is reported via a text string and progress bar. (3 and 4) After Question 9, the interface splits: The current question remains on the left (3); on the right, a table shows the data in their current state (4), which users can reference as they progress. (5) User inputs are validated in real time. Inadequate responses trigger warnings and disable the “Next” button (bottom of image) until corrected, largely automating QA/QC.

The “Submissions” tab guides contributors through submitting new surveys (Figure 2). While video/written tutorials are provided, the tab is designed to be self‐explanatory. Each question is validated in real time (Sievert et al. 2023); the “Next” button disables until required fields are adequately completed (Attali 2021), and invalid responses trigger informative warnings to help contributors resolve issues.

Throughout, the tab responds to the contributor's current situation and needs. For example, after a file is uploaded, the interface splits: one pane displays the current question while the other shows a preview of the data in their current state, allowing contributors to reference the data as they proceed. The tab also skips unnecessary questions to hasten submission. For example, if a contributor indicates their file includes point location data (Question #17), a follow‐up asking for a location file is skipped (Question #18). Once all questions are answered, users click “Submit,” and the app sends the raw, processed, location, and form‐response data to a Google Drive repository (Bryan et al. 2023; McGowan et al. 2023; Bryan and Posit Software PBC 2023), where they are later retrieved for post‐processing and QA/QC as described below. Importantly, submission is very flexible; the app's codebase is able to recognize many common data formats and adjust accordingly. For example, while PI data are generally stored with taxonomic data in columns, a user can trigger the app to transpose the data during submission if they have instead stored them in rows. If, at any point, the app fails to recognize the format of the user's data, submission arrests, and the app will inform the user about formatting expectations.

The tab's codebase also prioritizes three additional tasks: (1) metadata integration, (2) flagging possible errors, and (3) data standardization. PI survey data are more leverageable when combined with certain metadata not always included within survey files. Several questions solicit and merge in these metadata. For example, the tab asks for surveyors' names (Question #4), units for water‐depth data (Question #7), and the maximum value for the rake‐derived abundance data (Question #8).

Manual QA/QC can be prohibitively time‐consuming; because contributors typically understand their data best, we designed *P.I. Charter* to automate a considerable amount of QA/QC by leveraging the contributor's inputs. For example, the tab compares each column name in the submitted file to previously encountered names via a manually curated lookup table. Unrecognized names get flagged, prompting users to clarify them. This prevents unnecessary columns from entering the database. As another example, if a column name is ambiguous (e.g., “zp” could refer to *Zannichellia palustris* or *Zizania palustris* ), the lookup table will trigger the app to flag it as such for the user to clarify so it may be corrected during post‐submission QA/QC (see below).

Many during‐submission validation checks focus on the abundance data. Since these are typically numeric, any text (e.g., “Visual”) values are flagged so that the contributor can explain them as needed. Additionally, survey files often include rows of summary statistics and/or placeholder rows for unsampled locations. The app provides questions to help contributors identify and remove any such rows. Throughout, the app records all its “decisions” and caches the original submission file for reference, so no validation action is irreversible.

Despite considerable during‐submission validation, submitted data do not immediately enter the database. Instead, a successful submission triggers Google Drive to notify the app's maintainer. They then initiate a largely automated QA/QC and post‐processing pipeline. Briefly, this pipeline retrieves each submission in turn, runs additional checks, and presents summaries and views of each file for manual curation and correction by the maintainer. Only once a submission is approved is it then bound to the database file. At the end, an R script is sourced to produce all required summaries of the updated database file, and a new version of the app bundled with these files is pushed to the hosting platform. The process leverages R's *readline* function to largely preclude the need for any coding; approvals generally take a few minutes per submission while allowing for the human supervision needed to prevent errant data from entering the database. Interested readers are directed to the app's Github repository, where this process is extensively documented.

It is also noteworthy that the entire database is a single parquet file written using *arrow* (Richardson et al. 2026). Despite the file being > 300 columns by > 550,000 rows at time of writing, it is only a few megabytes in size, allowing us to bundle a copy with the app's other files for deployment to the hosting platform rather than hosting and accessing it separately, as would otherwise generally be done. This, coupled with most summarizations occurring during post‐processing rather than on demand in the app, allows the app to retrieve and display data to users very rapidly, enhancing the user experience.

### 2.4 Records Tab

**FIGURE 3:**
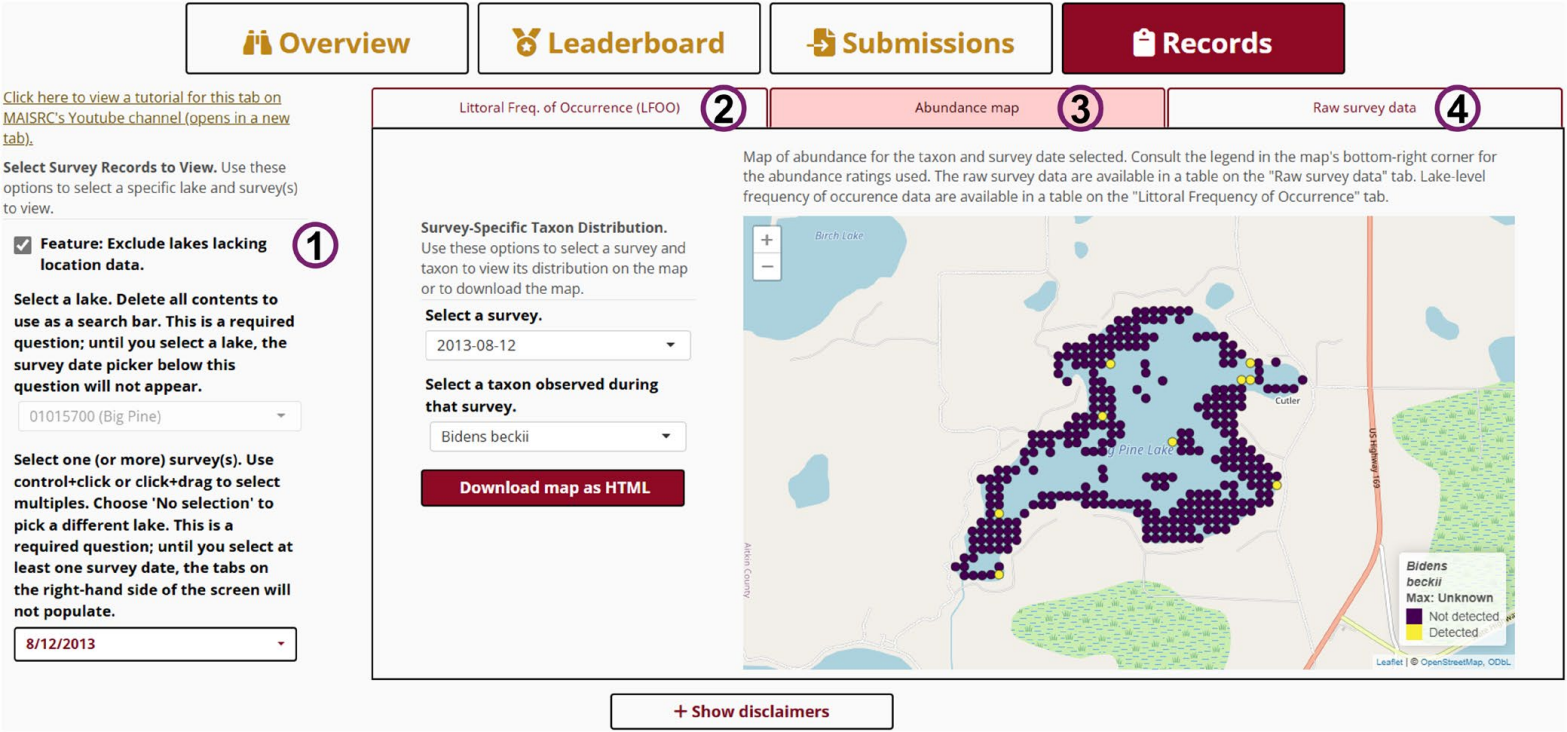
“Records” tab of *P.I. Charter*. The sidebar panel allows users to select lakes and surveys (1). The right panel includes three subtabs (2–4). (2) First (not shown) displays littoral frequency of occurrence (LFOO) data for all taxa for all selected surveys. (3) Second (shown) presents a map of taxon‐specific abundance data for the selected survey. (4) Third (not shown) displays raw data from the selected survey(s) in a tabular, downloadable format.

The “Records” tab is designed to share and recontextualize the data in the database (Figure 3). It has a sidebar for selecting specific surveys and a main panel displaying outputs across three subtabs. In the sidebar, users select a lake and then specific surveys conducted there. A checkbox allows users to exclude surveys lacking point‐level location data. On the basis of their selections, the app generates summaries and visualizations for display.

The first subtab displays littoral frequency of occurrence (LFOO, i.e., the percent of sampled points occupied) for all observed taxa as a *plotly* (Sievert 2020) bar graph (for one survey) or line graph showing change over time (for multiple surveys). The second subtab presents the raw tabular survey data from the database. Users can download these for offline use along with a guide to the database's columns. The third subtab displays a map of point‐level abundance data for a given survey and taxon (provided location data are available).

To respect data sensitivity and sovereignty, some restrictions are placed on this and the “Overview” tabs. First, lakes adjacent to Tribal reservations cannot be accessed. Second, protected species, i.e., those with state or federal designation as species of concern, are redacted. Lastly, certain personal information, such as contributors' email addresses and names of Minnesota Department of Natural Resources surveyors, are redacted. Rationale for these decisions is provided in the “Disclaimers” menus on the respective tabs.

### 2.5 Leaderboard Tab

The “Leaderboard” tab is designed to celebrate and reward our contributors. It features two tables: one listing contributors and another listing surveyors, along with the numbers of surveys associated with each. This tab acknowledges the countless hours spent by surveyors in the field, celebrates their diversity, and encourages continued data collection and submission.

We wish to acknowledge a few other tools used by *P.I. Charter*, as these may aid others wishing to build similar applications:

- *tidyverse* (Wickham et al. 2019) empowers the app's data‐wrangling and submission operations.
- *waiter* (Coene et al. 2022) supplies custom spinner elements on startup and elsewhere.
- *readxl* (Wickham and Bryan 2025) enables Excel file submission.
- *sf* (Pebesma and Bivand 2023) powers spatial operations for *leaflet* maps and enables shapefile submission.
- *shinydisconnect* (Attali 2023) makes the app's disconnect screen more informative.
- *viridis* (Garnier et al. 2024) provides visually accessible color palettes for visualizations.
- *shinyWidgets* (Perrier et al. 2025) provides feature‐rich multiple‐choice inputs.

We also emphasize our commitment to digital accessibility with *P.I. Charter*. As of June 2025, the app meets WCAG 2.1AA standards (https://www.w3.org/TR/WCAG21/) to the best of our abilities. Achieving compliance required concerted effort, reflecting our commitment to inclusivity.

Finally, we wish to address the app's name. “*P.I. Charter*” is superficially a play on “pie chart” and emphasizes the “P.I.” in PI surveying, but the name has two more symbolic components. First, it acknowledges the app as a literal charter—a collection of charts facilitating exploration. Second, it acknowledges the app is a figurative charter—an accord between us and our contributors to collectively build something more powerful than any of us could alone.

## 3 Results

From its debut in January 2023 to May 2026, *P.I. Charter* has received 1120 approved submissions from 45 different submitting entities, representing 135,091 point‐level observations on top of the > 300,000 previously manually compiled by Verhoeven et al. (2026) prior to the app's conception. New surveys spanned 325 different lakes sampled between 1999 and 2026. The app's shortened URL (z.umn.edu/PICharter), created in March 2023, has been accessed > 2775 times, and, since Google Analytics integration in February 2024, the app has recorded > 1400 engaged sessions from > 750 unique users. Partner reception to the app—including submission—has been consistently positive. Several contributors have left unsolicited comments describing submission as “easy,” “user‐friendly,” and “not time‐consuming.” One indicated that, through their experience, they learned ways to make their data cleaner in the future.

Contributors have been generally able to submit surveys quickly. Metadata from three recent submission batches—constituting 8, 15, and 9 surveys, respectively—showed average submission times of 100, 247, and 188 s per survey, respectively (including submitter downtime between submissions), although these data do not include any onboarding or pre‐processing the users may have performed prior to submission and may thus reflect the experience of more experienced users. We have also heard from users that they have adopted the contract language provided.

To further demonstrate the functionality and value of the app and its database, we next present four case studies (Boxes 1, 2, 3, 4) showcasing some of the types of questions they could help answer.

BOX 1 **How can past surveys guide future surveys?**

Lake plant communities are regularly surveyed in Minnesota, USA to detect invasive species, assess disturbance impacts, and monitor health. To interpret these data, however, managers must compare them with benchmarks—either to reference systems or, ideally, to the same lake in the past. Unfortunately, such data are often inaccessible, unfindable, or not interoperable (Phillips et al. 2016), particularly when different entities survey the same lake independently.

*P.I. Charter* helps address this problem. To illustrate, we walk through a real submission of a point‐intercept (PI) survey of Red Rock Lake (Hennepin County) and how *P.I. Charter* could have retroactively improved it.

**FIGURE 4:**
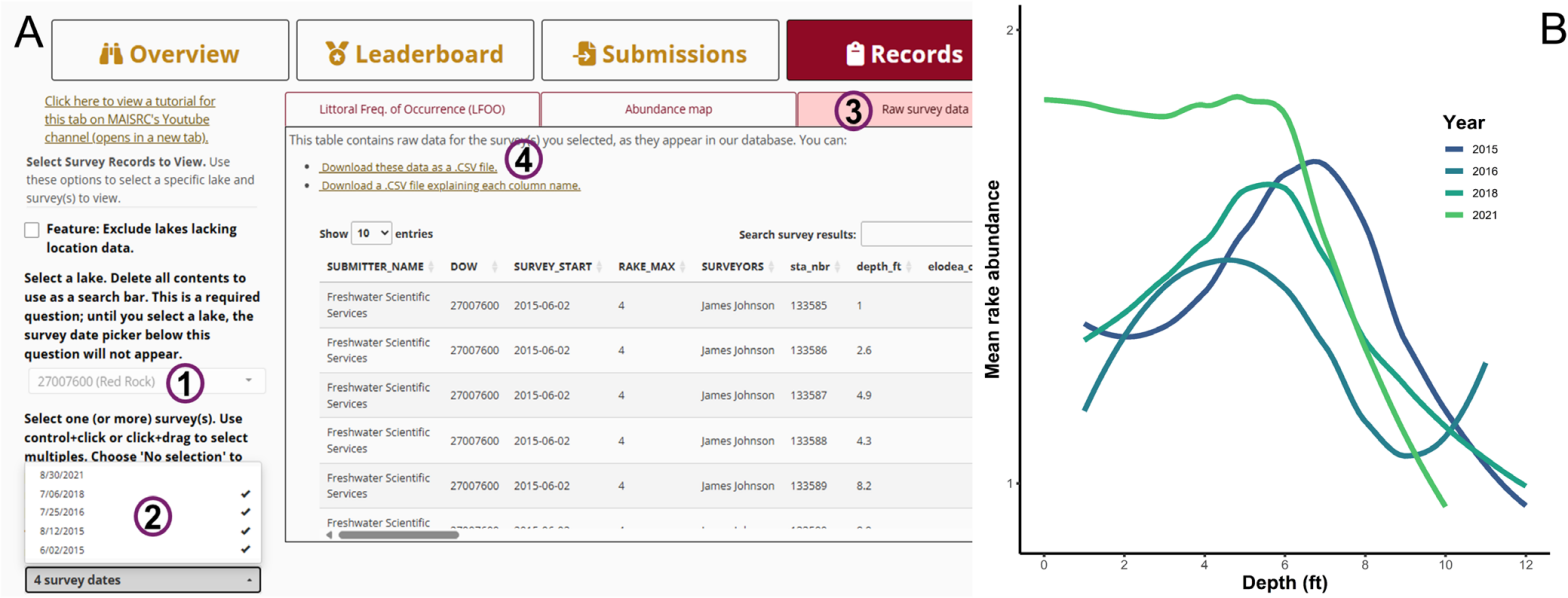
*P.I Charter* allows surveyors to quickly find existing, comparable data. (A) On the “Records” tab, a user can select a lake (1), then select any number of surveys from that lake (2). On the “Raw survey data” subtab (3), they can download the data (4) from those surveys. (B) Example of an analysis performable using the downloaded data: Water depth (feet; x‐axis) versus mean macrophyte abundance (y‐axis) at Red Rock Lake in Minnesota, USA over time (colors).

Before the survey, the surveyor could now begin on *P.I. Charter*'s “Overview” tab (Figure 2). Filtering records by lake, they could locate Red Rock Lake (DOW #27007600) to discover three prior surveys (from 2015 to 2018) they may have been unaware of. They could then have the app generate a list of all previously observed taxa in the lake (as well as in nearby lakes), helping them anticipate likely encounters and flag new occurrences that could warrant vouchering. They could then use the “Records” tab (Figure 4A) to view sampling grids used by past surveyors for increased comparability.

Then, after the survey, the manager could return to the “Submissions” tab to upload their data. The app would validate and clean their data set, ensuring interoperability with the existing database. From there, the manager could download the processed copy along with data from all prior surveys.

At this point, any analysis software could be used to compare past and present data. Figure 4B shows one example: a comparison of plant density by depth across years, a common output in monitoring reports. Because the underlying data are now interoperable, though, many other metrics and analyses would be feasible.

Previously, if historical surveys had been identified by a manager, often only the summary reports from those surveys might be available. Now, *P.I. Charter* exposes the raw data so managers can analyze them, produce relevant summaries, and use them to inform future survey efforts.

BOX 2 **Can snowfall be used to predict curly‐leaf pondweed coverage?**

Curly‐leaf pondweed (CLP; *Potamogeton crispus* ) is an invasive macrophyte common in Minnesota lakes. Previous studies have linked greater winter snow depth to reduced CLP abundance in spring, likely due to shading (Valley and Heiskary 2012; Verhoeven, Larkin, et al. 2020). While these two studies—spanning 11 and 50 lakes over 4 and 10 years, respectively—observed this general pattern, they relied on detailed, lake‐specific climatological data.

Meanwhile, managers often base spring management decisions on more accessible metrics like total regional snowfall. If this metric could reliably predict CLP abundance, managers could anticipate above‐average seasons for CLP, allocate limited resources more efficiently, and better communicate treatment expectations with stakeholders.

Here, we demonstrate how a manager could use data from *P.I. Charter*'s database and public snowfall data to examine relationships between CLP and snowfall. We compiled CLP littoral frequency of occurrence (LFOO) data from 629 spring surveys (March–June) across 158 lakes from Anoka, Carver, Dakota, Hennepin, Ramsey, Scott, and Washington Counties from *P.I. Charter*'s database and regional snowfall data from the Minnesota State Climatology Office (https://www.dnr.state.mn.us/climate/twin_cities/snowfall.html). We first assessed the relationship between CLP LFOO and snowfall from 2006 to 2015 (as in Verhoeven, Larkin, et al. 2020), then expanded the analysis to all available data between 1999 and 2023. Finally, we estimated lake‐specific slopes and *R* ^2^ values to evaluate how reliably snowfall predicted lake‐level CLP abundance.

**FIGURE 5:**
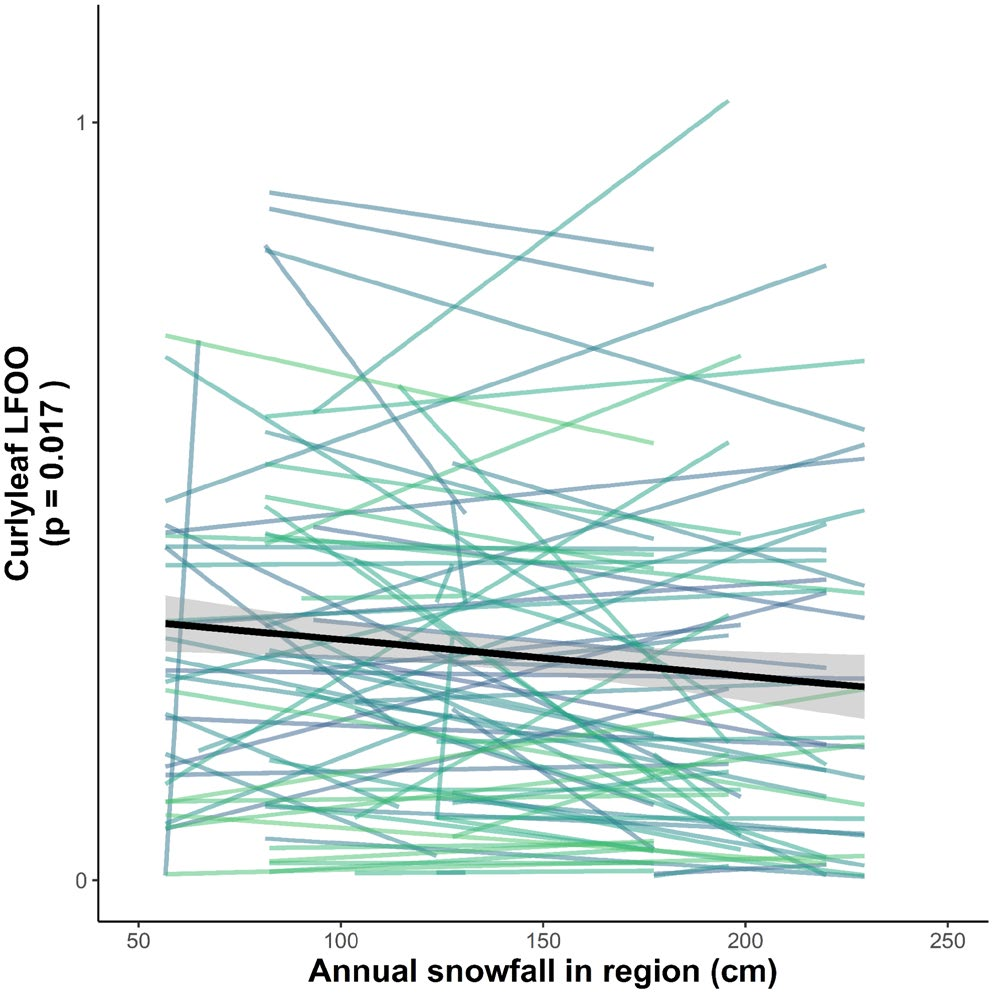
Overall relationship (black line, gray 95% Confidence Interval) between snowfall and curlyleaf pondweed (*Potamogeton crispus*) littoral frequency of occurrence (LFOO) is negative (*p* = 0.017), but individual lakes (*n* = 68) from the seven counties of the Twin Cities Metropolitan Region in Minnesota, USA showed mixed relationships (colored lines). Snowfall data are from the State Climatology Office. Curlyleaf pondweed LFOO data are from the *P.I. Charter* database from the same region.

Across the region, CLP LFOO was negatively associated with snowfall for both time frames (both *p* values < 0.05), ostensibly indicating snowfall can be used in place of snow depth. However, relationships varied widely by lake (Figure 5). Of the 68 lakes with ≥ 3 years of data, only 10 demonstrated strongly negative relationships (*R* ^2^ > 0.5). This suggests the relationship is not consistent enough to be used to guide management at the lake scale.

These findings highlight how *P.I. Charter*'s database can support both hypothesis testing and adaptive lake‐specific management. Managers may indeed find it useful to evaluate snow cover–CLP relationships for their lakes but should not over‐rely on regional snowfall data for this purpose because daily snow cover (depth) data—though harder to acquire—are more reliably predictive.

BOX 3 **Can we more efficiently hunt an emerging invasive threat?**

Hybrid watermilfoil (*Myriophyllum spicatum × sibiricum*), a cross between the native *M. sibiricum* and non‐native *M. spicatum* , is an increasingly problematic invasive macrophyte in the Midwestern U.S. (Moody and Les 2007). Hybrid vigor makes its management especially difficult; it can resist some herbicides (LaRue et al. 2013), grow more aggressively than either parent (Glisson and Larkin 2021), and create persistent, dense surface mats. Surveillance is hampered by its cryptic nature—it can only be confirmed via genetic testing—and by its spontaneous emergence wherever both parents co‐occur (Thum et al. 2020). Finding and discriminating hybrid watermilfoil is critical, especially when selecting herbicides.

**FIGURE 6:**
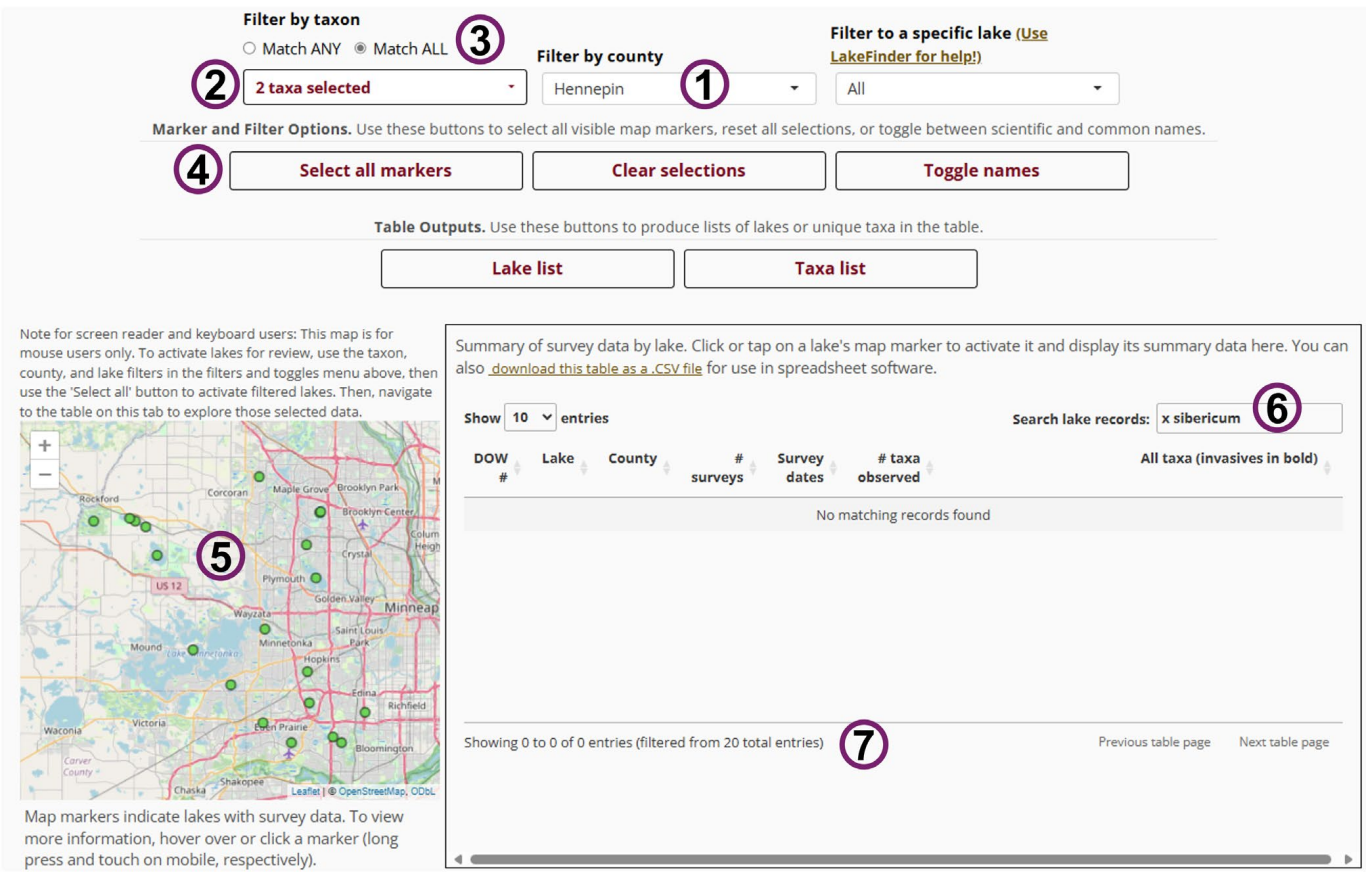
*P.I. Charter* allows users to filter the database by county (1) and taxon (2). Here, we filtered to lakes containing both *Myriophyllum spicatum* and *M. sibiricum* using the “Match all” option (3) in Hennepin County, Minnesota, USA. Then, users can activate all shown markers (4 and 5), loading their summary records. They can then search for strings in these summaries such as “*x sibiricum*” (6) to discover that no lakes, at time of writing, had observed populations of hybrid milfoil in our database (7).

One practical use of *P.I. Charter* is to identify lakes at risk of spontaneous emergence not yet known to have hybrid watermilfoil, such as those with records of both parents. To illustrate, consider a hypothetical invasive species manager working in Hennepin County, Minnesota, USA. Using the “Overview” tab, they filter records to those from Hennepin and find 112 lakes with records (as of June 2025; Figure 6). Next, they filter by taxon, selecting both *M. sibiricum* (29 lakes) and *M. spicatum* (45 lakes), then apply the “match all taxa” filter to identify 20 lakes where both species have been observed. They select all these and examine the associated summaries, noting that none, as yet, are known to contain hybrid watermilfoil, indicating they are prime candidates for genetic tests.

The manager could then use the “Records” tab to inspect past abundance maps (Figure 3) for each lake to identify areas the parent species might co‐occur and to search for *Myriophyllum* specimens with intermediate morphology and/or to collect tissue for genetic testing (Wolfe et al. 2025).

BOX 4 **How are Minnesota lake macrophyte communities changing?**

Non‐native macrophytes can become over‐abundant in lakes, causing ecological and economic harms. While management aims to reduce impacts on native species, it remains unclear how native species respond over time to invasions and to subsequent management of them.

The *P.I. Charter* data set spans over two decades and includes abundance data for both native and non‐native macrophytes. Many lakes now have time series long enough to examine trends in abundance and diversity of both groups. Here, we focus on two metrics: (1) changes in abundance of non‐native taxa and (2) changes in community diversity, measured using the Inverse Simpson's Diversity Index.

We filtered *P.I. Charter*'s database to lakes with at least 5 years of data collected between May and August (*N* = 139 as of May 7, 2025). For each lake, we modeled trends over time using generalized linear models: community diversity was modeled with a Tweedie distribution and log link; non‐native littoral frequency of occurrence (LFOO) was modeled with a Beta distribution and logit link using the *mgcv* package in R (Wood 2011).

**FIGURE 7:**
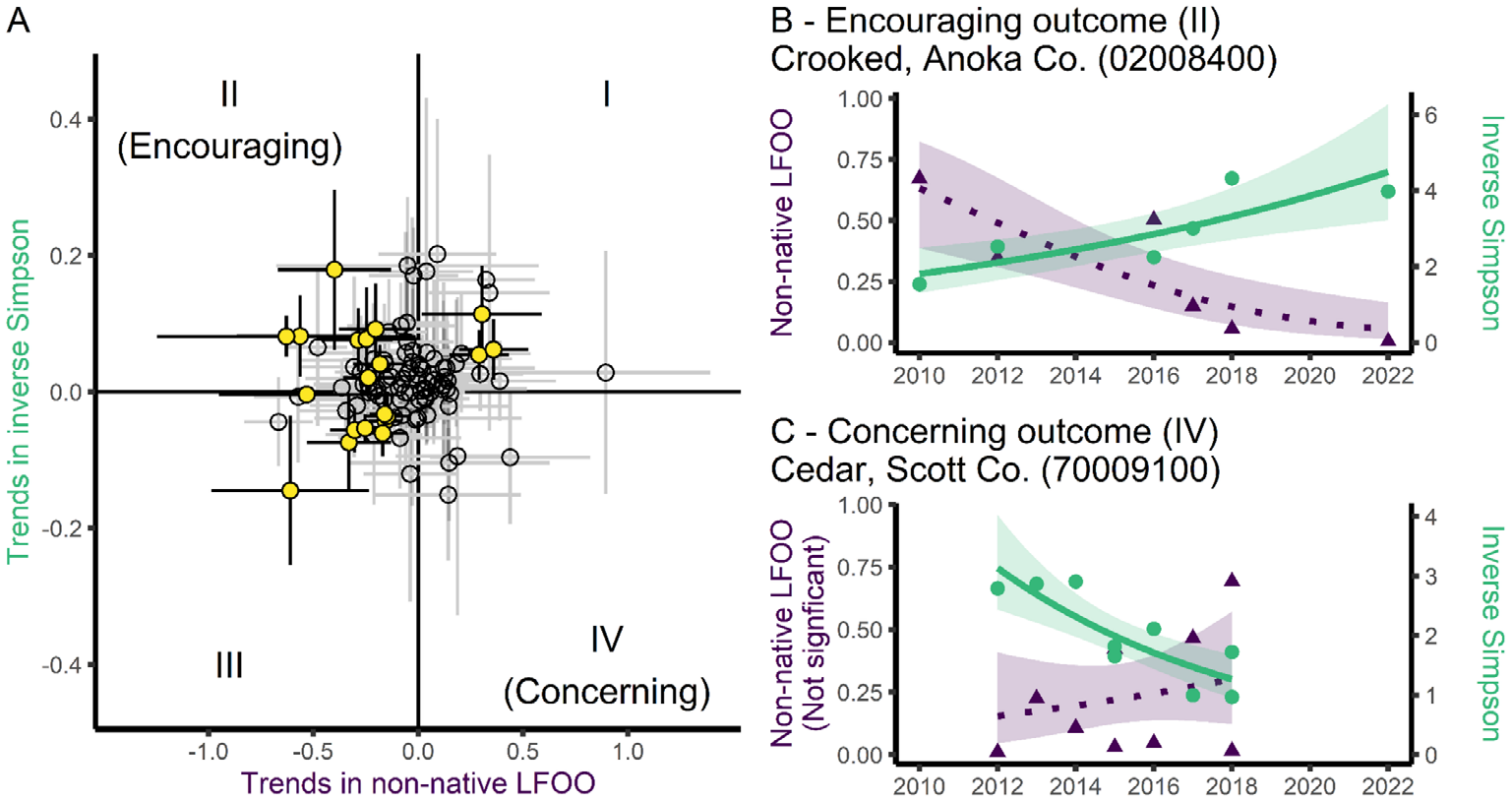
(A) Trends in macrophyte diversity (Inverse Simpson's Diversity Index; y‐axis) and non‐native abundance (littoral frequency of occurrence, “LFOO;” x‐axis) from 104 Minnesota, USA lakes with slope estimates for both metrics. Yellow points are lakes with significant trends for both metrics. Gray and black lines represent 95% Confidence Intervals. (B) Example of an encouraging trajectory: Macrophyte diversity has increased over time and non‐native abundance has decreased (Crooked Lake in Anoka County). (C) Example of a concerning trajectory: Macrophyte diversity has declined over time and non‐native abundance has increased nonsignificantly (Cedar Lake in Scott County). Points in panels B and C are raw data while lines are trend lines with shaded 95% Confidence Intervals. The eight‐digit values in parentheses are Minnesota‐specific identifiers.

Figure 7A shows each lake's trend in overall macrophyte diversity against its trend in non‐native species coverage (*N* = 104 lakes with trend estimates for both metrics). This bivariate plot enables classification of lakes by management trajectory—for example, quadrant II shows increasing diversity and decreasing non‐native abundance (an encouraging trajectory), whereas quadrant IV shows decreasing diversity and increasing non‐native abundance (a potential management concern).

Notably, many lakes (47%) showed no significant change in either metric (Figure 7A). Among the 104 lakes with slope estimates for both metrics, only 20 showed significant trends for both. Of these, 8 fell into quadrant II—i.e., improved macrophyte diversity alongside reduced non‐native abundance (Figure 7B). In contrast, none showed the opposite trend (Figure 7C shows an example with a nonsignificant increase in non‐native LFOO).

These findings underscore the value of long‐term data for evaluating macrophyte community change. While most lake monitoring is *ad hoc*, *P.I. Charter* enables broader‐scale assessment of native and non‐native species dynamics. The insights gleaned could inform adaptive management, highlight where further investigation is warranted, and guide strategy development at local, regional, and statewide scales.

## 4 Discussion

*P.I. Charter* is a new Shiny web application that solicits, tidies, compiles, visualizes, and disseminates point‐intercept macrophyte survey data. We have demonstrated that the app has received substantial uptake, is meeting goals around ease of use, accessibility, and education, and shows potential to answer a range of ecological questions.

However, beyond its specific uses, *P.I. Charter* also exemplifies the untapped potential of apps in contexts where data are gathered and/or housed disparately. *P.I. Charter* is one of the few web applications we have seen to combine data submission, largely automated QA/QC, data compilation, and public access. While other Shiny apps we have found have some of these components, none have them all. For example, *ContDataQC* (Pennino et al. 2025) accepts continuous sensor data, aggregates compatible records, and supports QA/QC, but does not perform QA/QC itself nor compile submissions. *CWDAT* (Gray et al. 2021) allows users to upload water‐quality data and performs validation but focuses on analysis facilitation and does not generate a public database. Several apps for behavioral data described in Colomb and Winter (2021) provide for cleaning and formatting but are not web‐hosted, and submission to a central repository is separate and optional. Wisconsin's *Aquatic Plant Explorer* (Wisconsin Department of Natural Resources 2025) serves Wisconsin PI data provided to the agency but does not receive and collate the data nor perform QA/QC.

As such, *P.I. Charter* may be the first of its kind—but it should not be the last. Any research collective, interjurisdictional group, or citizen‐science program that gathers high‐value data could benefit from an app like *P.I. Charter* that can handle submission, QA/QC, standardization, compilation, visualization, and sharing. These apps can not only unlock new insights and analytical opportunities, but—as our experience shows—encourage interoperability in data storage and methodology too (Kasprzak et al. 2020).

However, while Shiny offers many advantages for building science‐focused apps, we encountered notable challenges building *P.I. Charter*. Its advantages include broad familiarity with R among ecologists (Li 2020), Shiny's native inclusion of semantic HTML tags (Jia et al. 2022), integration with Google Analytics (Whitehead and Booker 2019) and GitHub (Kaufman 2020) for code version control, and Shiny's many widget and layout options (Kaufman 2020). It is also open‐source and free (Pennino et al. 2025). However, Shiny currently has a steep learning curve even for those with considerable R familiarity (Kasprzak et al. 2020), limited and unintuitive debugging tools (Jia et al. 2022), few flexible hosting options (Li 2020), and potential security risks (Kasprzak et al. 2020). It is also difficult to support long‐term hosting and maintenance on soft funding (Whitehead and Booker 2019). In our case, these challenges were mitigated by having dedicated development staff with significant programming experience, long‐term funding, and few data‐security concerns. That combination may be rare; wider adoption of Shiny will ultimately require its developers to further address these barriers.

We also did not personally experience some of Shiny's cited advantages. For example, while Shiny is praised for requiring minimal HTML, CSS, or JavaScript expertise (Jia et al. 2022), we found substantial knowledge of these languages essential, even with support from AI agents. *P.I. Charter*'s user interface has required hundreds of lines of CSS, HTML, and JavaScript to enable non‐native behaviors, enhance user experience, and improve accessibility. Accessibility was a particular challenge. We found Shiny's native elements regularly fell short of WCAG 2.1AA standards, e.g., many widgets lack appropriate ARIA attributes, modals and notifications do not manage focus correctly, and keyboard navigability is inconsistent. Given ethical and legal imperatives around accessibility, building compliant apps currently requires significant development effort and experience.

Performance concerns, by contrast, were largely addressable. *P.I. Charter* is a great counterexample to the misconception that Shiny cannot construct complex, data‐heavy apps (Jia et al. 2022; Kasprzak et al. 2020). We found the following practices helpful: modularizing each tab's code into separate user‐interface and server files, restricting development to one tab at a time, pre‐generating large objects and loading them only on demand, and using proxy functions to update user‐interface elements efficiently. We also stored our data set as a parquet file, as previously mentioned. In our view, many of Shiny's perceived limitations are not inherent but rather stem from underuse of its extended ecosystem (see Jia et al. 2022; Gibbons et al. 2023; Li 2020). This work serves as an example of how greater adoption of existing tools and workarounds can overcome the aforementioned limitations.

*P.I. Charter* addresses several areas of need in aquatic plant ecology and management. From a basic ecology perspective, fundamental aspects of macro‐ and community ecology are underdeveloped in aquatic vis‐a‐vis terrestrial ecosystems (Alahuhta et al. 2021). A critical gap *P.I. Charter* could address is answering how environmental and biotic factors interact to shape community structure (Alahuhta et al. 2025). The app's species‐occurrence data can be readily linked to lake environmental data sets. Occurrence data across multiple spatial scales (from point to lake to regional) can be used to investigate relationships between community structure and abiotic variables (Box 3). The availability of point‐level data, delineating co‐occurrence (or lack thereof) of species at the scale at which plants interact, is particularly powerful (Box 4). These fine‐scale data distinguish *P.I. Charter* from other global macrophyte syntheses (e.g., Murphy et al. 2019) and could be used to infer species interactions or other ecological dynamics (Alahuhta et al. 2025).

*P.I. Charter* is also suitable for answering applied questions. For example, the possible impacts of invasive plants on native species can be explored (Box 4). With a planned future integration of treatment records, the effectiveness of invasive plant management could also be evaluated (e.g., Verhoeven, Larkin, et al. 2020). If sufficient data are accumulated, it may be possible for resource managers to identify monitoring data from lakes with comparable features to a lake targeted for new management to establish better‐informed baseline expectations. For example, plant community change in managed lakes in the same ecoregion, of similar size and depth to the target lake, could be examined to set realistic management targets.

A common question we have heard is: “Why is a research institution (MAISRC) hosting *P.I. Charter*?” Indeed, *Aquatic Plant Explorer* is hosted by the Wisconsin Department of Natural Resources (WI DNR 2025). In our case, it proved more feasible, timely, and low‐risk for MAISRC to host the app than for any other entity to do so. Unlike Wisconsin—where the DNR has long encouraged submissions to a statewide database following specific survey and data‐reporting protocols (Hauxwell et al. 2010)—Minnesota lacks a state‐organized system for submitting PI surveys. MAISRC also had a head start in building such a database: existing partnerships with surveyors from past research projects (Verhoeven, Glisson, et al. 2020; Verhoeven, Larkin, et al. 2020) provided early buy‐in, raw survey files for testing and development, and collaborators willing to try and provide feedback on the app. A nongovernmental entity with no enforcement or compliance mechanism might also be seen as a safer aggregator by some contributors. Finally, hosting *P.I. Charter* aligns closely with MAISRC's mission to inspire action by making decision‐relevant data accessible.

*P.I. Charter* embraces the ideal that open data sharing is valuable. Data accessibility is increasingly being prioritized to bolster trust and transparency (Colomb and Winter 2021) and facilitate effective management (Glisson et al. 2025). While full accessibility is well‐intentioned, we nevertheless must consider that withholding data can be justifiable. For example, in compliance with statutes, we de‐identify species of conservation concern and, to respect data sovereignty of Tribal Nations, we have hidden all data from lakes intersecting Tribal reservation boundaries. While these decisions may cause *P.I. Charter*'s visualizations to sometimes underrepresent the data available, we argue preventing potential ecological, legal, and cultural harms takes precedence. We encourage others building similar platforms to work closely with contributors and impacted stakeholders to understand potential unintended consequences before making data available.

Looking ahead, we plan to expand *P.I. Charter* to new areas, contributors, and visualization spaces. We are conversing with teams in other states about making all our data interoperable to enable adding additional states in the future, greatly increasing the scale of questions and audiences the database could serve. Second, although the app already has many contributors and is promoted through biannual outreach to hundreds more, we know additional, uncompiled data still exist. For example, several consultants have expressed interest in contributing but have not yet done so. We plan to increase outreach to ensure the database is as comprehensive as possible. Finally, we intend to expand our visualization suite. In particular, we are currently developing displays related to aquatic plant management cataloged by the Minnesota DNR, which could help better contextualize observed changes in plant communities over time. With continued engagement and refinement, *P.I. Charter* is poised to advance basic and applied work in aquatic plant ecology and, we hope, serve as a model for apps in other, similar contexts.

## Author Contributions

**Alex W. Bajcz:** conceptualization (lead), data curation (lead), methodology (lead), project administration (lead), software (lead), supervision (lead), visualization (supporting), writing – original draft (lead), writing – review and editing (lead). **Daniel J. Larkin:** formal analysis (lead), investigation (lead), methodology (supporting), project administration (supporting), supervision (supporting), writing – original draft (supporting), writing – review and editing (supporting). **Michael R. Verhoeven:** conceptualization (supporting), data curation (supporting), formal analysis (lead), investigation (lead), resources (equal), visualization (supporting), writing – original draft (supporting), writing – review and editing (supporting). **Raymond M. Newman:** formal analysis (equal), investigation (lead), project administration (supporting), resources (equal), writing – original draft (supporting), writing – review and editing (supporting). **Jake R. Walsh:** conceptualization (supporting), data curation (supporting), formal analysis (equal), investigation (lead), resources (equal), visualization (supporting), writing – original draft (supporting), writing – review and editing (supporting). **Abha Panda:** formal analysis (equal), investigation (equal), visualization (supporting), writing – original draft (supporting), writing – review and editing (supporting). **Nicholas B. D. Phelps:** conceptualization (supporting), funding acquisition (lead), project administration (supporting), supervision (supporting), writing – original draft (supporting), writing – review and editing (supporting).

## Funding

This work was supported by the Minnesota Aquatic Invasive Species Research Center (MAISRC) and State of Minnesota.

## Conflicts of Interest

The authors declare no conflicts of interest.

## Data Availability

All nonsensitive data in *P.I. Charter's* database are available via the app's Records tab; the app can be accessed at the persistent link https://z.umn.edu/PICharter. The data are also available by request from the app's maintainer. The app's codebase (with a few redactions related to sensitive data) is persistently available on the app's Github page: https://github.com/MAISRC/PICharter. This includes extensive documentation on the during‐ and post‐submission data validation and QA/QC processes applied to new submissions before they enter the database.

## References

[ece373779-bib-0052] Alahuhta, J. , J. García‐Girón , E. Molina‐Navarro , and K. Murphy . 2025. “Freshwater Plant Macroecology Needs to Step Forward From the Shadows of the Terrestrial Domain.” Nordia Geographical Publications 54: 1–9. 10.30671/nordia.149042.

[ece373779-bib-0001] Alahuhta, J. , M. Lindholm , L. Baastrup‐Spohr , et al. 2021. “Macroecology of Macrophytes in the Freshwater Realm: Patterns, Mechanisms and Implications.” Aquatic Botany 168: 103325. 10.1016/j.aquabot.2020.103325.

[ece373779-bib-0002] Alexander, C. 2022. Procedure for Aquatic Vegetation Surveys. Michigan Department of Environment, Great Lakes, and Energy. https://www.michigan.gov/egle/‐/media/Project/Websites/egle/Documents/Policies‐Procedures/WRD/WRD‐PS‐006‐Aquatic‐Vegetation‐Surveys.pdf.

[ece373779-bib-0003] Attali, D. 2021. “shinyjs: Easily Improve the User Experience of Your Shiny Apps in Seconds (Version 2.1.0) [Computer Software].” https://cran.r‐project.org/web/packages/shinyjs/index.html.

[ece373779-bib-0004] Attali, D. 2023. “shinydisconnect: Show a Nice Message When a “Shiny” App Disconnects or Errors (Version 0.1.1) [Computer Software].” https://cran.r‐project.org/web/packages/shinydisconnect/index.html.

[ece373779-bib-0005] Borgelt, J. , M. Dorber , M. A. Høiberg , and F. Verones . 2022. “More Than Half of Data Deficient Species Predicted To Be Threatened by Extinction.” Communications Biology 5, no. 1: 679. 10.1038/s42003-022-03638-9. 35927327 PMC9352662

[ece373779-bib-0006] Bryan, J. , C. Citro , H. Wickham , Google Inc , and Posit Software PBC . 2023. “gargle: Utilities for Working With Google APIs (Version 1.5.2) [Computer Software].” https://cran.r‐project.org/web/packages/gargle/index.html.

[ece373779-bib-0007] Bryan, J. , and Posit Software PBC . 2023. “googlesheets4: Access Google Sheets Using the Sheets API V4 (Version 1.1.1) [Computer Software].” https://cran.r‐project.org/web/packages/googlesheets4/index.html.

[ece373779-bib-0008] Chang, W. , J. Cheng , J. Allaire , et al. 2026. “_shiny: Web Application Framework for R_.” 10.32614/CRAN.package.shiny.

[ece373779-bib-0009] Cheng, J. , B. Schloerke , B. Karambelkar , et al. 2025. “leaflet: Create Interactive Web Maps With the JavaScript “Leaflet” Library (Version 2.2.2) [Computer Software].” https://cran.r‐project.org/web/packages/leaflet/index.html.

[ece373779-bib-0010] Coene, J. , J. Kim , and V. Granda . 2022. “waiter: Loading Screen for ‘Shiny’ (Version 0.2.5) [Computer Software].” https://cran.r‐project.org/web/packages/waiter/index.html.

[ece373779-bib-0011] Colomb, J. , and Y. Winter . 2021. “Creating Detailed Metadata for an R Shiny Analysis of Rodent Behavior Sequence Data Detected Along One Light‐Dark Cycle.” Frontiers in Neuroscience 15: 742652. 10.3389/fnins.2021.742652. 34899155 PMC8661901

[ece373779-bib-0012] Garnier, S. , N. Ross , R. Rudis , A. P. Camargo , M. Sciaini , and C. Scherer . 2024. “viridis(Lite)—Colorblind‐Friendly Color Maps for R (Version 0.6.5) [Computer Software].”

[ece373779-bib-0013] Gibbons, A. , I. Donohue , C. Gorman , E. King , and A. Parnell . 2023. “NEAL: An Open‐Source Tool for Audio Annotation.” PeerJ 11: e15913. 10.7717/peerj.15913. 37645015 PMC10461540

[ece373779-bib-0014] Glisson, W. J. , and D. J. Larkin . 2021. “Hybrid Watermilfoil (* Myriophyllum spicatum × Myriophyllum sibiricum *) Exhibits Traits Associated With Greater Invasiveness Than Its Introduced and Native Parental Taxa.” Biological Invasions 23, no. 8: 2417–2433. 10.1007/s10530-021-02514-7.

[ece373779-bib-0015] Glisson, W. J. , M. Nault , C. Jurek , et al. 2025. “Evaluation of a Decade of Management of a North American Aquatic Invasive Species (*Nitellopsis obtusa*) Highlights Scale‐Dependent Effectiveness and Monitoring Gaps.” Facets 10: 1–14. 10.1139/facets-2024-0104.

[ece373779-bib-0016] Gray, A. , C. Robertson , and R. Feick . 2021. “CWDAT—An Open‐Source Tool for the Visualization and Analysis of Community‐Generated Water Quality Data.” ISPRS International Journal of Geo‐Information 10, no. 4: 207. 10.3390/ijgi10040207.

[ece373779-bib-0017] Hauxwell, J. , S. Knight , K. Wagner , A. Mikulyuk , M. Nault , and S. Chase . 2010. Recommended Baseline Monitoring of Aquatic Plants in Wisconsin: Sampling Design, Field and Laboratory Procedures, Data Entry and Analysis, and Applications (PUB‐SS‐1068). Wisconsin Department of Natural Resources Bureau of Science Services. https://www3.uwsp.edu/cnr‐ap/UWEXLakes/Documents/ecology/Aquatic%20Plants/PI‐Protocol‐2010.pdf.

[ece373779-bib-0018] Jia, L. , W. Yao , Y. Jiang , et al. 2022. “Development of Interactive Biological Web Applications With R/Shiny.” Briefings in Bioinformatics 23, no. 1: bbab415. 10.1093/bib/bbab415. 34642739

[ece373779-bib-0019] Kasprzak, P. , L. Mitchell , O. Kravchuk , and A. Timmins . 2020. “Six Years of Shiny in Research ‐ Collaborative Development of Web Tools in R.” R Journal 12, no. 2: 155. 10.32614/RJ-2021-004.

[ece373779-bib-0020] Kaufman, A. R. 2020. “Implementing Novel, Flexible, and Powerful Survey Designs in R Shiny.” PLoS One 15, no. 4: e0232424. 10.1371/journal.pone.0232424. 32353057 PMC7192460

[ece373779-bib-0021] LaRue, E. A. , M. P. Zuellig , M. D. Netherland , M. A. Heilman , and R. A. Thum . 2013. “Hybrid Watermilfoil Lineages Are More Invasive and Less Sensitive to a Commonly Used Herbicide Than Their Exotic Parent (Eurasian Watermilfoil).” Evolutionary Applications 6, no. 3: 462–471. 10.1111/eva.12027. 23745138 PMC3673474

[ece373779-bib-0022] Li, Y. 2020. “Towards Fast Prototyping of Cloud‐Based Environmental Decision Support Systems for Environmental Scientists Using R Shiny and Docker.” Environmental Modelling & Software 132: 104797. 10.1016/j.envsoft.2020.104797.

[ece373779-bib-0023] Madsen, J. D. , and R. M. Wersal . 2018. “Proper Survey Methods for Research of Aquatic Plant Ecology and Management.” Journal of Aquatic Plant Management 56s: 90–96.

[ece373779-bib-0024] McGowan, L. D. , J. Bryan , and Posit Software PBC . 2023. “googledrive: An Interface to Google Drive (Version 2.1.1) [Computer Software].” https://cran.r‐project.org/web/packages/googledrive/index.html.

[ece373779-bib-0025] McGuire, R. M. , K. T. Hayashi , X. Yan , et al. 2022. “EcoEvoApps: Interactive Apps for Theoretical Models in Ecology and Evolutionary Biology.” Ecology and Evolution 12, no. 12: e9556. 10.1002/ece3.9556. 36479028 PMC9719042

[ece373779-bib-0026] Michigan Department of Environment, Great Lakes, and Energy . 2022. Procedure for Aquatic Vegetation Surveys (WRD‐PS‐006). Michigan Department of Environment, Great Lakes, and Energy. https://www.michigan.gov/egle/‐/media/Project/Websites/egle/Documents/Policies‐Procedures/WRD/WRD‐PS‐006‐Aquatic‐Vegetation‐Surveys.pdf.

[ece373779-bib-0027] Moody, M. L. , and D. H. Les . 2007. “Geographic Distribution and Genotypic Composition of Invasive Hybrid Watermilfoil (* Myriophyllum spicatum × M. sibiricum *) Populations in North America.” Biological Invasions 9, no. 5: 559–570. 10.1007/s10530-006-9058-9.

[ece373779-bib-0028] Murphy, K. , A. Efremov , T. A. Davidson , et al. 2019. “World Distribution, Diversity and Endemism of Aquatic Macrophytes.” Aquatic Botany 158: 103127. 10.1016/j.aquabot.2019.06.006.

[ece373779-bib-0029] Pebesma, E. , and R. Bivand . 2023. Spatial Data Science: With Applications in R. 1st ed. Chapman and Hall/CRC. 10.1201/9780429459016.

[ece373779-bib-0030] Pennino, M. J. , J. Stamp , E. W. Leppo , D. A. Gibbs , and B. G. Bierwagen . 2025. “ContDataQC: An R Package and Shiny App for Quality Control of Continuous Water Quality Sensor Data.” SoftwareX 30: 102124. 10.1016/j.softx.2025.102124. 40546755 PMC12180758

[ece373779-bib-0031] Perleberg, D. , P. Radomski , S. Simon , K. Carlson , and J. Knopik . 2016. Minnesota Lake Plant Survey Manual, for Use by MNDNR Fisheries Section and EWR Lake Habitat Program. Minnesota Department of Natural Resources. Ecological and Water Resources Division.

[ece373779-bib-0032] Perrier, V. , F. Meyer , and D. Granjon . 2025. “shinyWidgets: Custom Inputs Widgets for Shiny (Version 0.9.0.9) [Computer Software].” https://github.com/dreamRs/shinyWidgets.

[ece373779-bib-0033] Phillips, G. , N. Willby , and B. Moss . 2016. “Submerged Macrophyte Decline in Shallow Lakes: What Have We Learnt in the Last Forty Years?” Aquatic Botany 135: 37–45. 10.1016/j.aquabot.2016.04.004.

[ece373779-bib-0034] R Core Team . 2025. R: A Language and Environment for Statistical Computing [Computer Software]. R Foundation for Statistical Computing. https://www.R‐project.org/.

[ece373779-bib-0035] Richardson, N. , I. Cook , N. Crane , et al. 2026. “_arrow: Integration to 'Apache' 'Arrow'_.” 10.32614/CRAN.package.arrow.

[ece373779-bib-0036] Sievert, C. 2020. Interactive Web‐Based Data Visualization With R, Plotly, and Shiny. 1st ed. Chapman and Hall/CRC. 10.1201/9780429447273.

[ece373779-bib-0037] Sievert, C. , R. Iannone , J. Cheng , and Posit Software PBC . 2023. “shinyvalidate: Input Validation for Shiny Apps (Version 0.1.3) [Computer Software].” https://cran.r‐project.org/web/packages/shinyvalidate/index.html.

[ece373779-bib-0038] Thum, R. A. , G. M. Chorak , R. M. Newman , et al. 2020. “Genetic Diversity and Differentiation in Populations of Invasive Eurasian ( *Myriophyllum spicatum* ) and Hybrid ( *Myriophyllum spicatum* × *Myriophyllum sibiricum* ) Watermilfoil.” Invasive Plant Science and Management 13, no. 2: 59–67. 10.1017/inp.2020.12.

[ece373779-bib-0039] Valley, R. D. , and S. Heiskary . 2012. “Short‐Term Declines in Curlyleaf Pondweed in Minnesota: Potential Influences of Snowfall.” Lake and Reservoir Management 28, no. 4: 338–345. 10.1080/07438141.2012.745911.

[ece373779-bib-0040] Verhoeven, M. R. , W. L. Bartodziej , M. S. Berg , et al. 2026. “Occurrence and Environmental Data for Aquatic Plants of Minnesota From 1999‐2018.” Scientific Data 13: 650. 10.1038/s41597-026-07027-3. 41813674 PMC13111723

[ece373779-bib-0041] Verhoeven, M. R. , W. J. Glisson , and D. J. Larkin . 2020. “Niche Models Differentiate Potential Impacts of Two Aquatic Invasive Plant Species on Native Macrophytes.” Diversity 12, no. 4: 162. 10.3390/d12040162.

[ece373779-bib-0042] Verhoeven, M. R. , D. J. Larkin , and R. M. Newman . 2020. “Constraining Invader Dominance: Effects of Repeated Herbicidal Management and Environmental Factors on Curlyleaf Pondweed Dynamics in 50 Minnesota Lakes.” Freshwater Biology 65, no. 5: 849–862. 10.1111/fwb.13468.

[ece373779-bib-0043] Welsh, A. H. , D. B. Lindenmayer , and C. F. Donnelly . 2013. “Fitting and Interpreting Occupancy Models.” PLoS One 8, no. 1: e52015. 10.1371/journal.pone.0052015. 23326323 PMC3542396

[ece373779-bib-0044] Whitehead, A. L. , and D. J. Booker . 2019. “Communicating Biophysical Conditions Across New Zealand's Rivers Using an Interactive Webtool.” New Zealand Journal of Marine and Freshwater Research 53, no. 2: 278–287. 10.1080/00288330.2018.1532914.

[ece373779-bib-0045] Wickham, H. , M. Averick , J. Bryan , et al. 2019. “Welcome to the Tidyverse.” Journal of Open Source Software 4, no. 43: 1686. 10.21105/joss.01686.

[ece373779-bib-0046] Wickham, H. , and J. Bryan . 2025. “_readxl: Read Excel Files_.” 10.32614/CRAN.package.readxl.

[ece373779-bib-0047] Wilkinson, M. D. , M. Dumontier , I. J. J. Aalbersberg , et al. 2016. “The FAIR Guiding Principles for Scientific Data Management and Stewardship.” Scientific Data 3: 160018. 10.1038/sdata.2016.18. 26978244 PMC4792175

[ece373779-bib-0048] Wisconsin Department of Natural Resources . 2025. “Aquatic Plant Explorer.” https://dnr‐wisconsin.shinyapps.io/AquaticPlantExplorer/.

[ece373779-bib-0049] Wolfe, A. L. , A. W. Bajcz , R. M. Newman , and R. A. Thum . 2025. “MilfoilMapper: A Web‐Based Tool to Inform Eurasian Watermilfoil ( *Myriophyllum spicatum* ) Management.” Invasive Plant Science and Management 18: e31. 10.1017/inp.2025.10032.

[ece373779-bib-0050] Wood, S. N. 2011. “Fast Stable Restricted Maximum Likelihood and Marginal Likelihood Estimation of Semiparametric Generalized Linear Models.” Journal of the Royal Statistical Society, Series B: Statistical Methodology 73, no. 1: 3–36. 10.1111/j.1467-9868.2010.00749.x.

[ece373779-bib-0051] Xie, Y. , J. Cheng , X. Tan , et al. 2024. “DT: A Wrapper of the JavaScript Library ‘DataTables’ (Version 0.33) [Computer Software].” https://cran.r‐project.org/web/packages/DT/.

